# Supplementary material for: Synthesis and Evaluation of Reactive Oxygen Species Sensitive Prodrugs of a NAMPT Inhibitor FK866
Source: Molecules. 2022 Dec 25;28(1):169. doi: 10.3390/molecules28010169 (PMC9821821; doi:10.3390/molecules28010169)

Synthesis and Evaluation of Reactive Oxygen Species Sensitive Prodrugs of a NAMPT Inhibitor FK866

Zili Xu [1,2,3,†], Huihui Wang [1,3,4,†], Haixia Liu [1,2,3], Hongli Chen [1,3,*] and Biao Jiang [1,3,*]

1 Shanghai Institute for Advanced Immunochemical Studies, ShanghaiTech University, 393 Middle Huaxia Road, Pudong, Shanghai 201210
2 School of Physical Science and Technology, ShanghaiTech University, 393 Middle Huaxia Road, Shanghai 201210, China
3 University of Chinese Academy of Sciences, 19A Yuquan Road, Shijingshan District, Beijing 100049, China
4 School of Life Science and Technology, ShanghaiTech University, 393 Middle Huaxia Road, Shanghai 201210, China
* Correspondence: chenhl@shanghaitech.edu.cn (H.C.); jiangbiao@shanghaitech.edu.cn (B.J.)
† These authors contributed equally to this work.

^{1}H NMR of **122-001**:

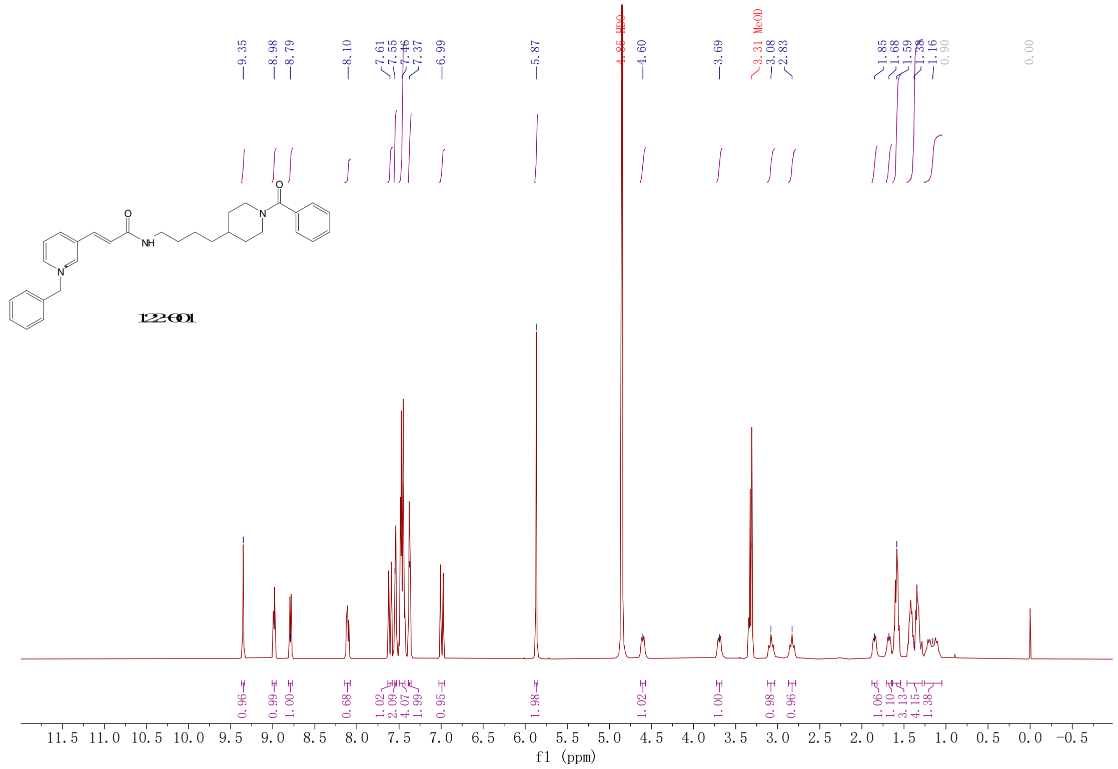

^{13}C NMR of 122-001

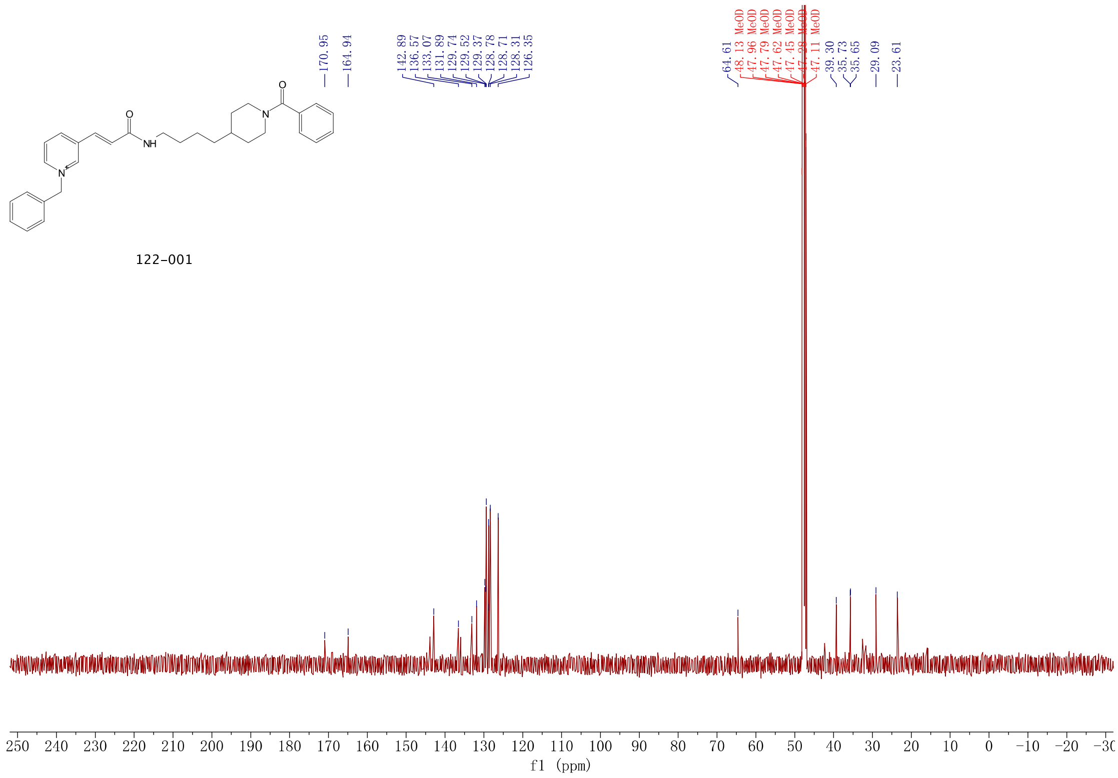

^{1}H NMR of **122-052**

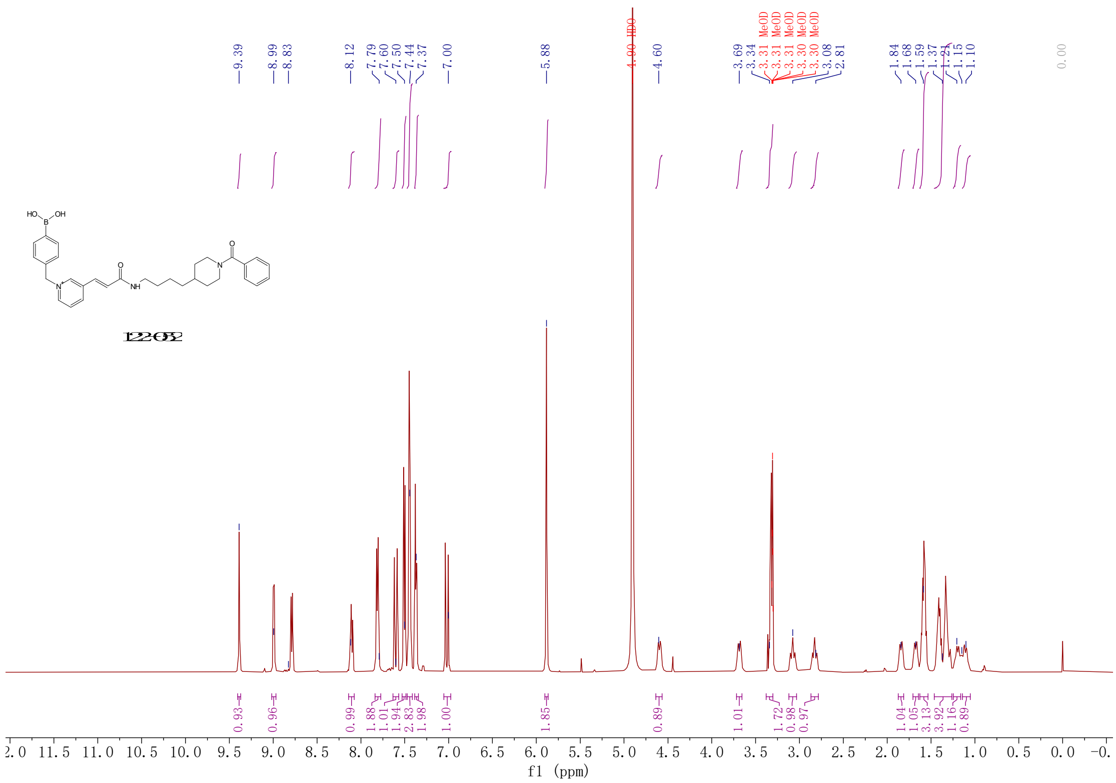

122-052

^{13}C NMR of **122-052**

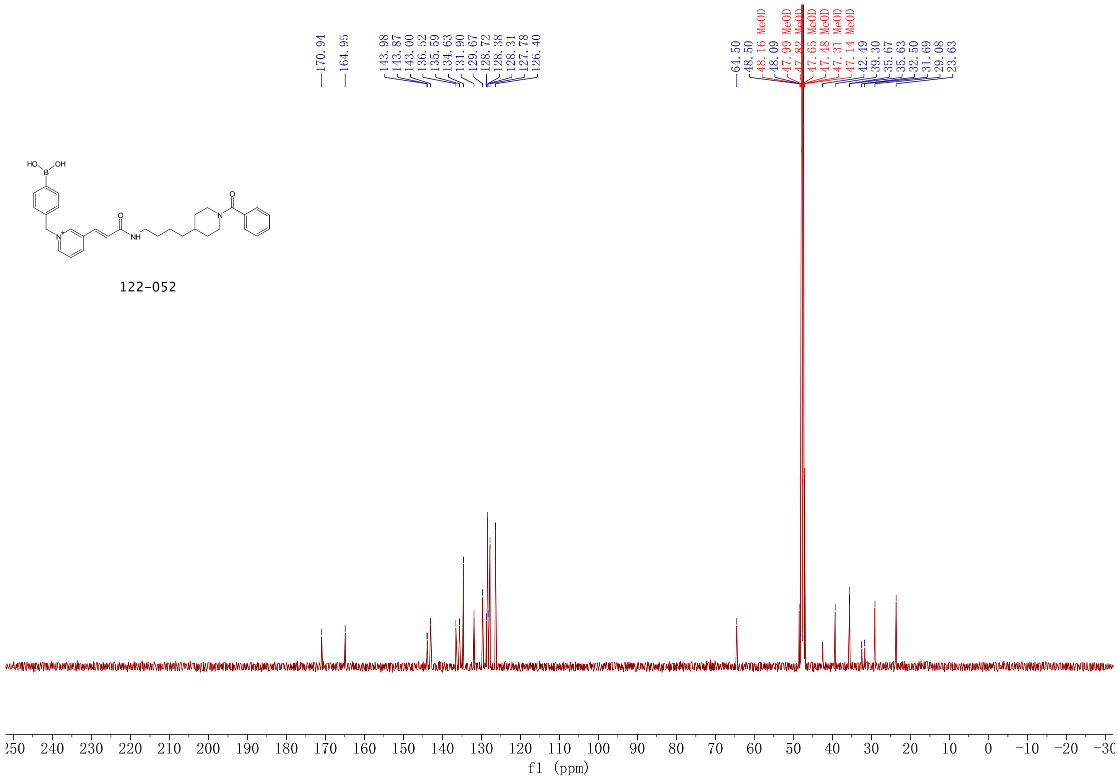

122-052

^{1}H NMR of compound **2**

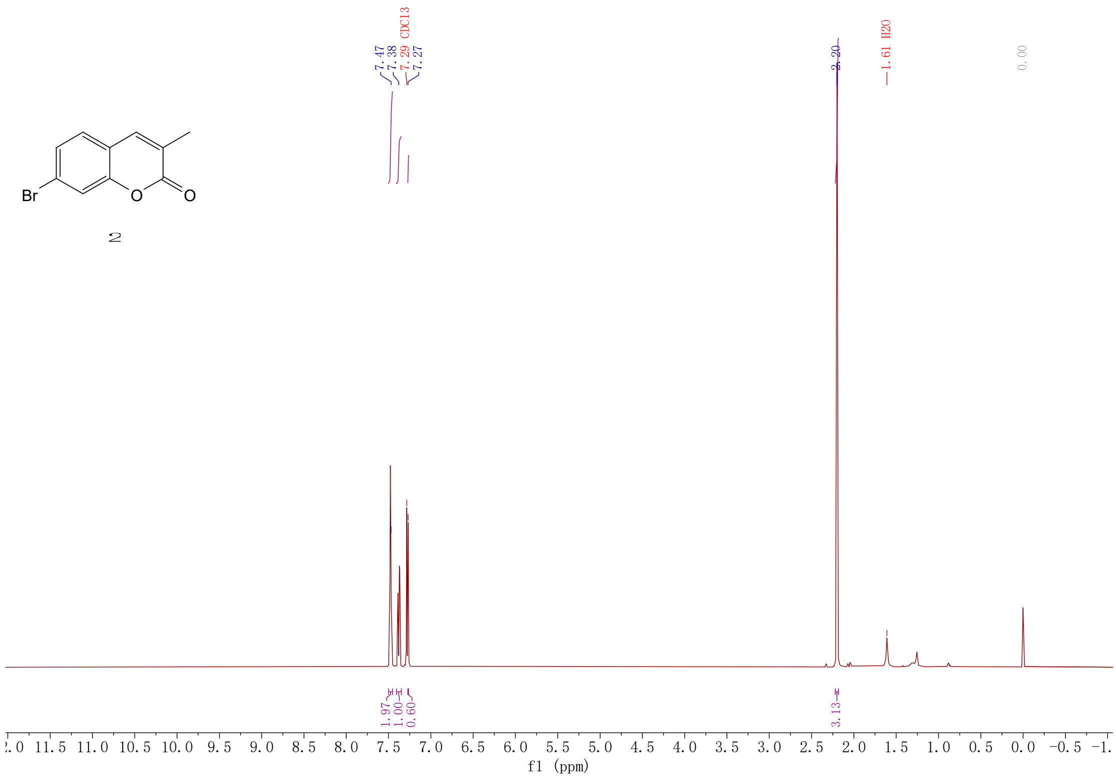

^{13}C NMR of compound 2

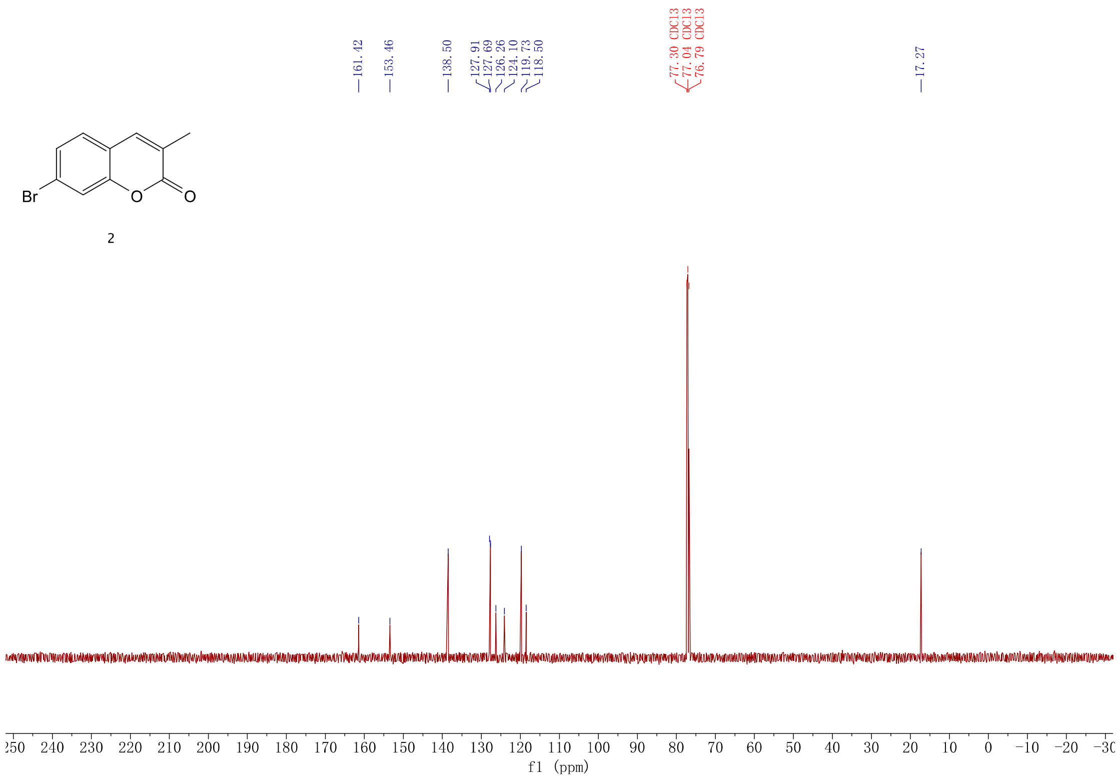

^{1}H NMR of compound **3**

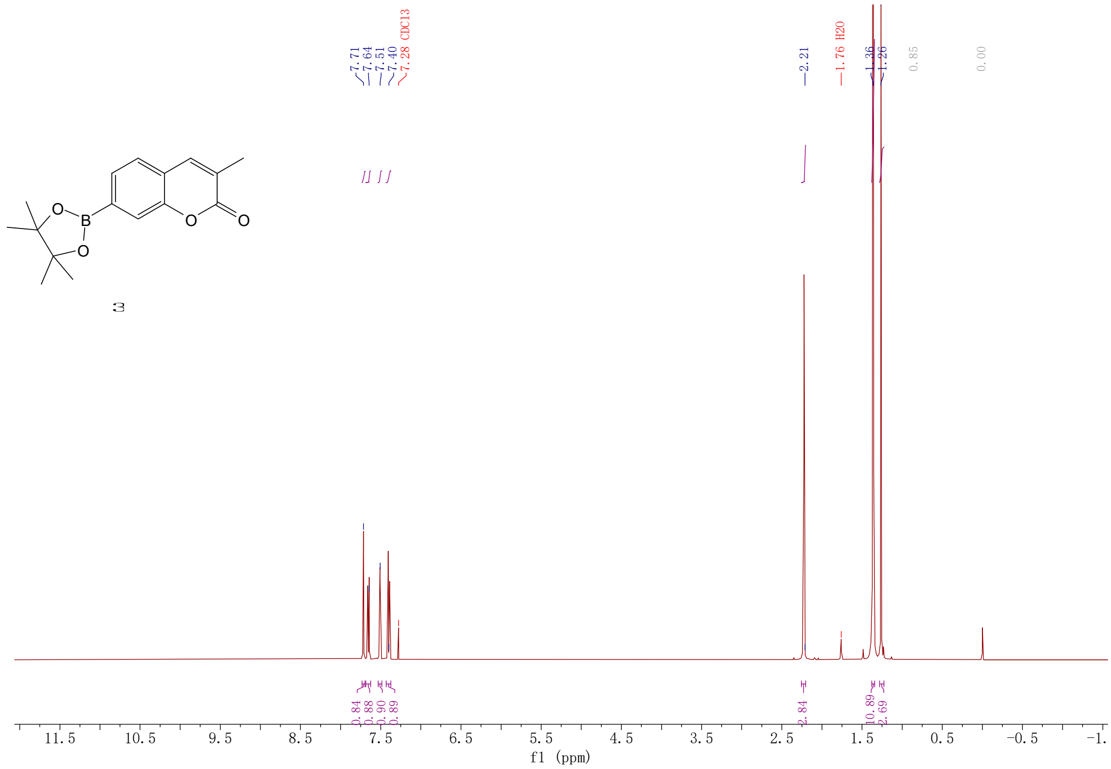

^{13}C NMR of compound **3**

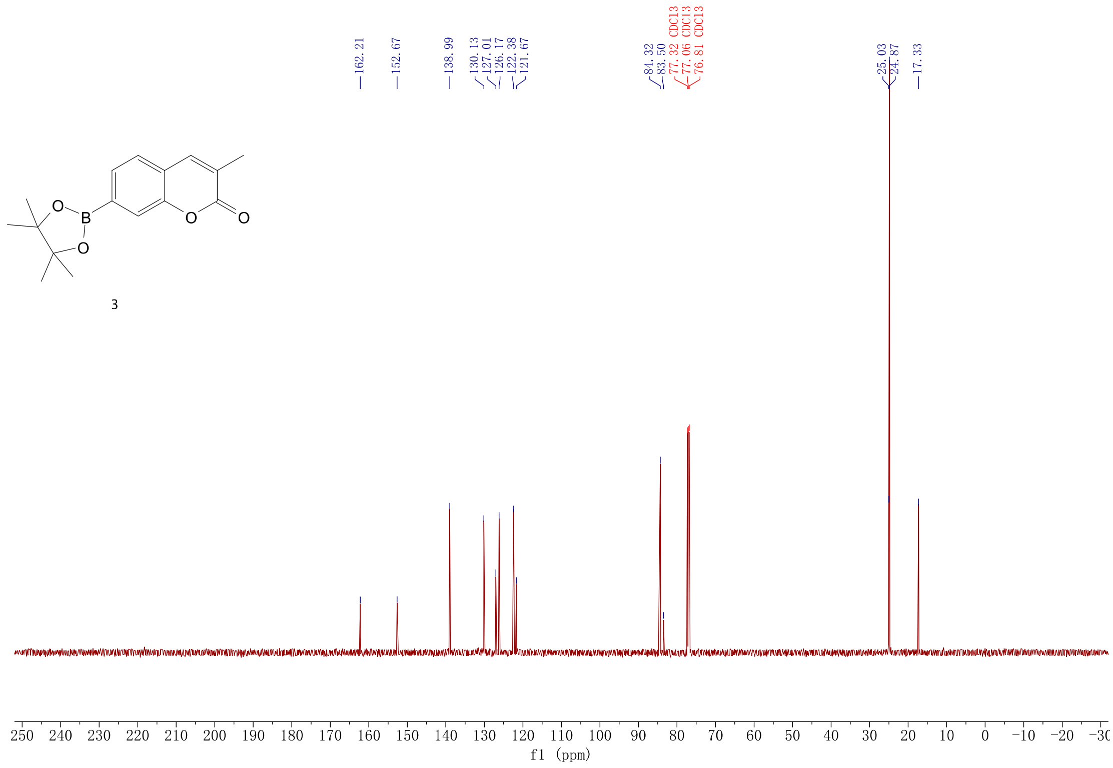

^{1}H NMR of compound **4**

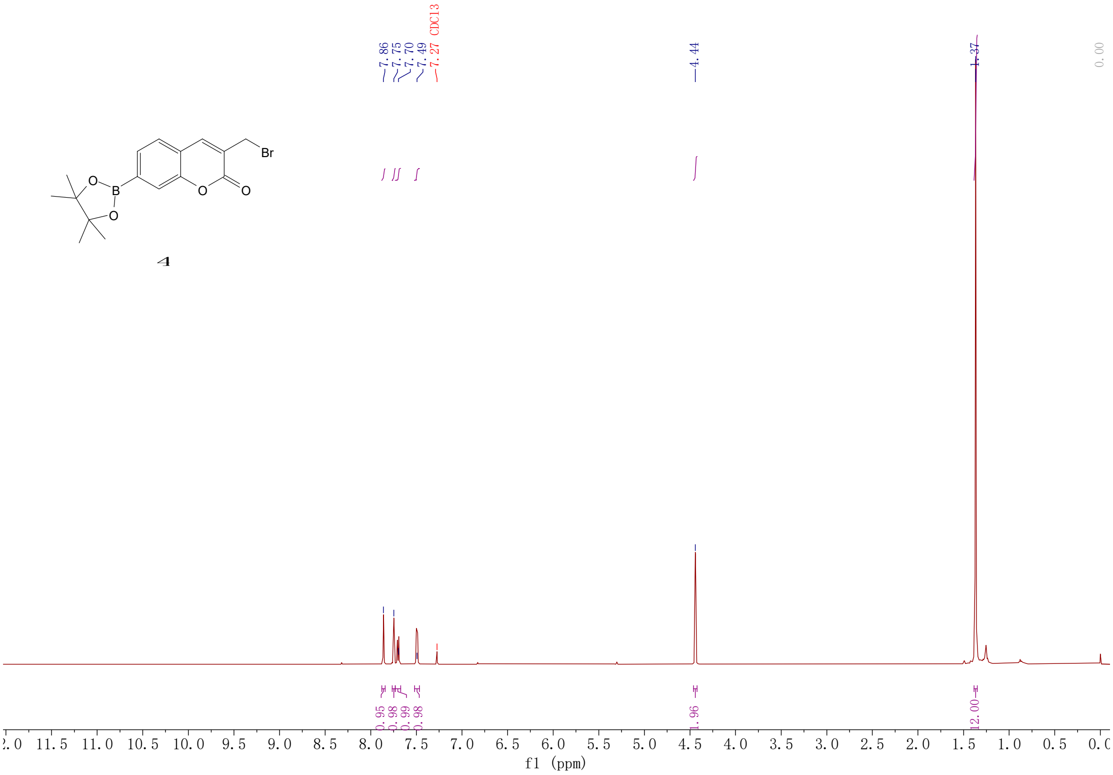

^{13}C NMR of compound **4**

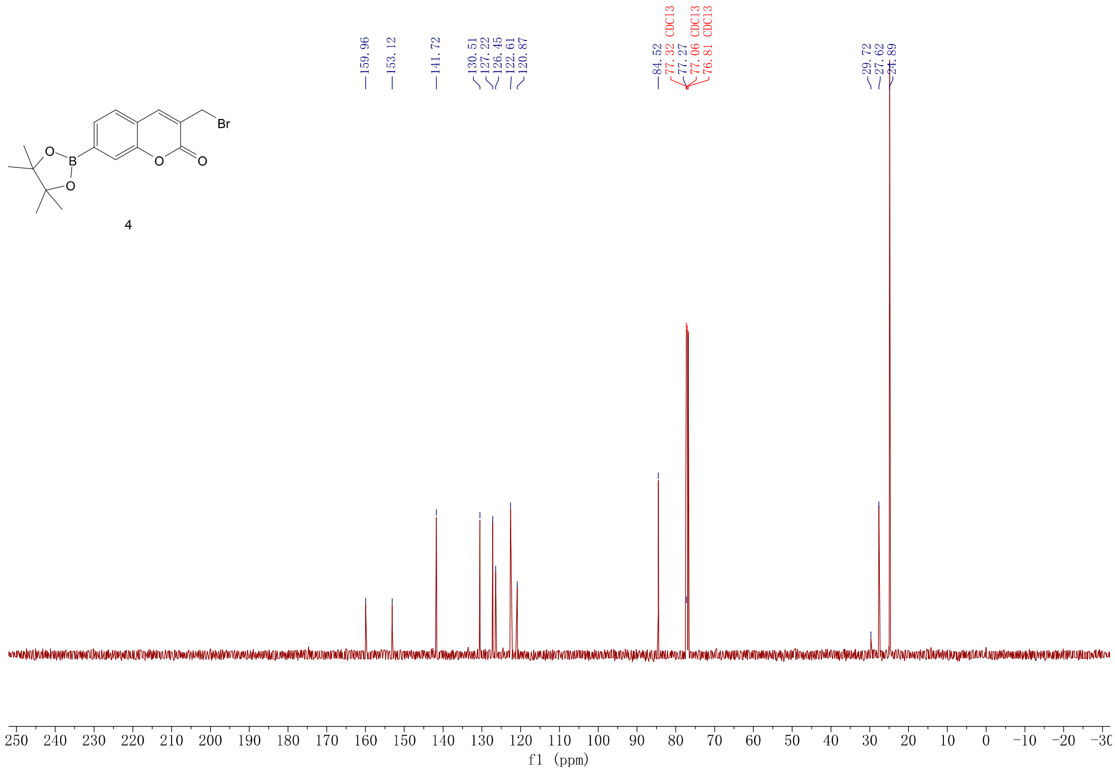

^{1}H NMR of **122-066**

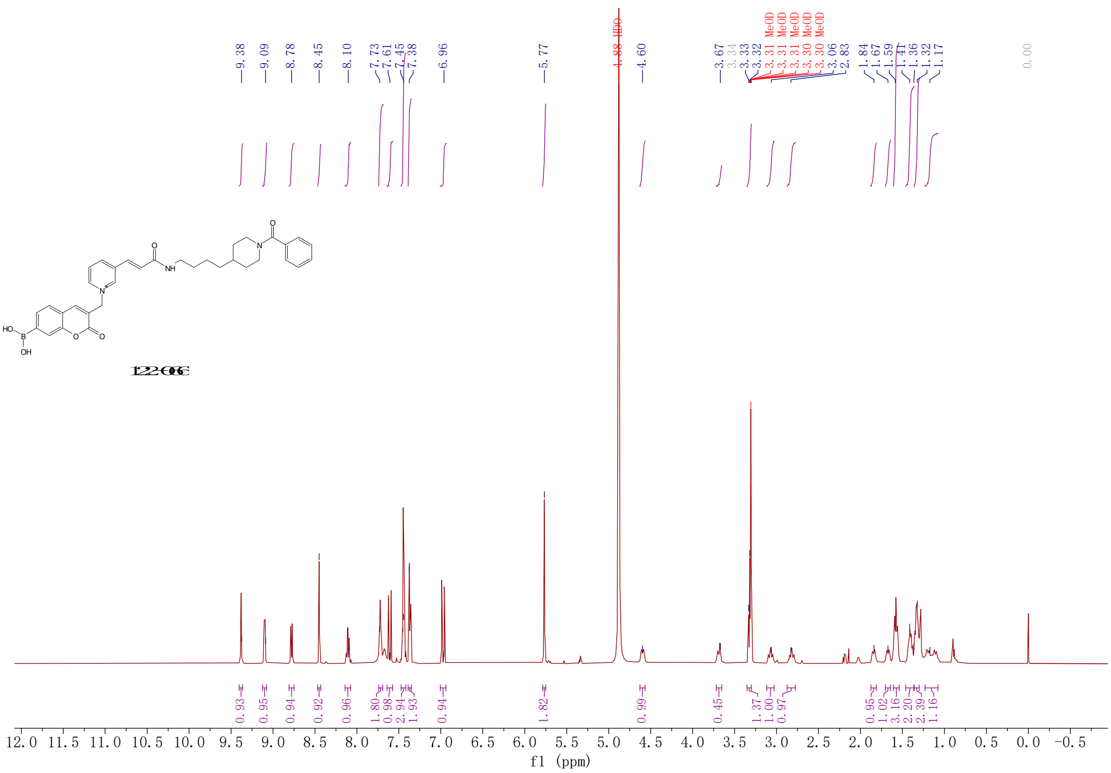

^{13}C NMR of **122-066**

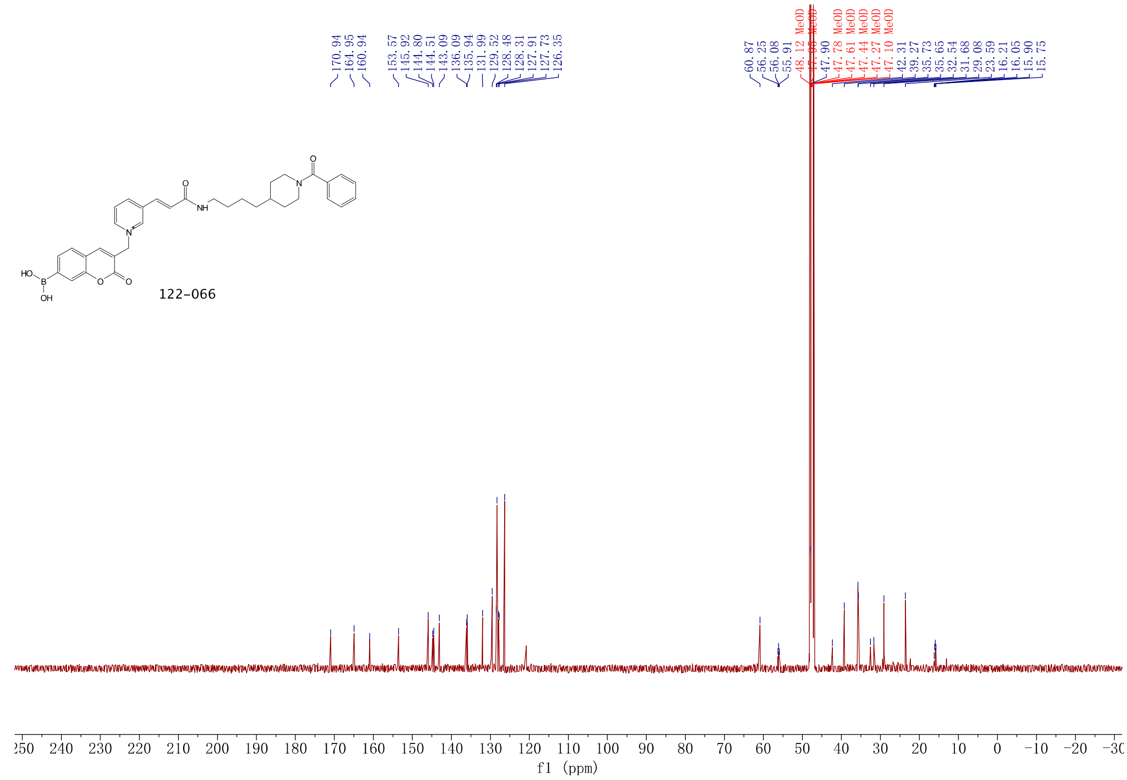

Supplement: Supplementary file 1 [file molecules-28-00169-s001.zip › molecules-2093278-SI.pdf]
